# HIRA complex deposition of histone H3.3 is driven by histone tetramerization and histone-DNA binding

**DOI:** 10.1016/j.jbc.2024.107604

**Published:** 2024-07-24

**Authors:** Austin Vogt, Mary Szurgot, Lauren Gardner, David C. Schultz, Ronen Marmorstein

**Affiliations:** 1 Department of Biochemistry and Biophysics, Perelman School of Medicine at the University of Pennsylvania, Philadelphia, Pennsylvania, USA; 2 Abramson Family Cancer Research Center, Perelman School of Medicine at the University of Pennsylvania, Pennsylvania, USA; 3 Graduate Group in Biochemistry and Molecular Biophysics, Perelman School of Medicine at the University of Pennsylvania, Pennsylvania, USA

**Keywords:** HIRA, UBN1, CABIN1, ASF1a, histone H3.3, histone deposition, chromatin regulation

## Abstract

The HIRA histone chaperone complex is comprised of four protein subunits: HIRA, UBN1, CABIN1, and transiently associated ASF1a. All four subunits have been demonstrated to play a role in the deposition of the histone variant H3.3 onto areas of actively transcribed euchromatin in cells. The mechanism by which these subunits function together to drive histone deposition has remained poorly understood. Here we present biochemical and biophysical data supporting a model whereby ASF1a delivers histone H3.3/H4 dimers to the HIRA complex, H3.3/H4 tetramerization drives the association of two HIRA/UBN1 complexes, and the affinity of the histones for DNA drives release of ASF1a and subsequent histone deposition. These findings have implications for understanding how other histone chaperone complexes may mediate histone deposition.

Cells rely on small, basic scaffolding proteins called histones to condense roughly 2 m of linear DNA into the nucleus (1, 2, 3, 4, 5, 6). In all, 147 base pairs of DNA associate with two copies each of core histones H2A, H2B, H3, and H4 upon replication to package the genetic material into nucleosomes-the simplest repeating subunit of chromatin (2, 3). More specifically, nucleosome formation proceeds first by two H3/H4 dimers joining to form a tetramer that is deposited onto about 80 base pairs of DNA to form the tetrasome, which is then flanked by two H2A/H2B dimers and the remaining 60 bp of DNA to complete the nucleosome (4).

The deposition of histones is coordinated by a group of protein complexes collectively known as histone chaperones. Histone chaperones are widely disparate in sequence, structure, and size, but are generally all able to shield histones from aberrant electrostatic interactions with nucleic acids and proteins and to interact with other nuclear machinery to coordinate deposition to the appropriate genomic locations (5, 6, 7, 8). Specific types of nuclear machinery with which different histone chaperones must interact include the nuclear pore (9), centromeric (10), replication (11), and transcriptional complexes (12, 13). During periods of active transcription, for example, core histones must be evicted to access the underlying DNA and are replaced with variant histone H3.3 chaperoned by the HIRA complex to mediate transcriptional activity at a given gene locus (14, 15, 16, 17).

Among the H3/H4- specific histone chaperones identified, most have been shown to have functionally relevant multimerization states that assist in tetrasome assembly on or near target DNA (18, 19, 20, 21, 22). For example, HJURP monomers chaperone the H3 variant CENP-A-containing histone dimers to the centromere. HJURP homodimerizes *via* a C-terminal motif to drive two CENP-A/H4 dimers together to form a centromeric tetrasome (18). The yeast H3/H4 chaperone Rtt106 has both a dimerization domain and an additional histone coordination domain that recognizes H3K56 acetylation (19), and the dimerization domain likely drives H3/H4 tetramer formation before deposition (20). The CAF1 complex, which chaperones canonical histone H3.1/H4 dimers and couples histone deposition to DNA replication, is a heterotrimeric complex containing an autoinhibited DNA binding domain that is released upon H3.1/H4 binding. CAF1 dimerization is then induced through the binding of two histone-loaded CAF1 complexes to a DNA substrate and deposition of tetrameric H3.1/H4 follows (21, 22, 23, 24).

The current understanding of the HIRA histone chaperone complex includes some similarities to these other H3/H4 chaperones, but the functional relevance of each subunit in the context of tetrasome formation is unclear. First, we know that ASF1a binds the tetramerization interface of H3/H4 heterodimers upon their translation in the cytosol, delivers them to the nucleus, and effectively sequesters them as dimers until they have been delivered to the correct nuclear location where handoff to a subsequent histone chaperone proceeds (25, 26).

We have previously identified UBN1 as the subunit responsible for (1) HIRA complex binding specificity to variant histone H3.3 over the canonical H3.1 through a Hpc2 Related Domain (HRD) (27), (2) tightly binding to and stabilizing HIRA’s N-terminal WD40 repeat domain through a N-terminal to HRD domain (NHRD) (28), and (3) electrostatically associating with double-stranded DNA (29) (Fig. 1). The crystal structure of a UBN1/H3.3/H4 complex revealed that HRD binding to H3.3 could theoretically accommodate either histone heterodimers or heterotetramers but would occlude the DNA binding surface on the histones in either case (27). A structured region in the middle of UBN1 (middle domain) has also been reported to form a homodimer *in vitro* (29).

**Figure 1:**
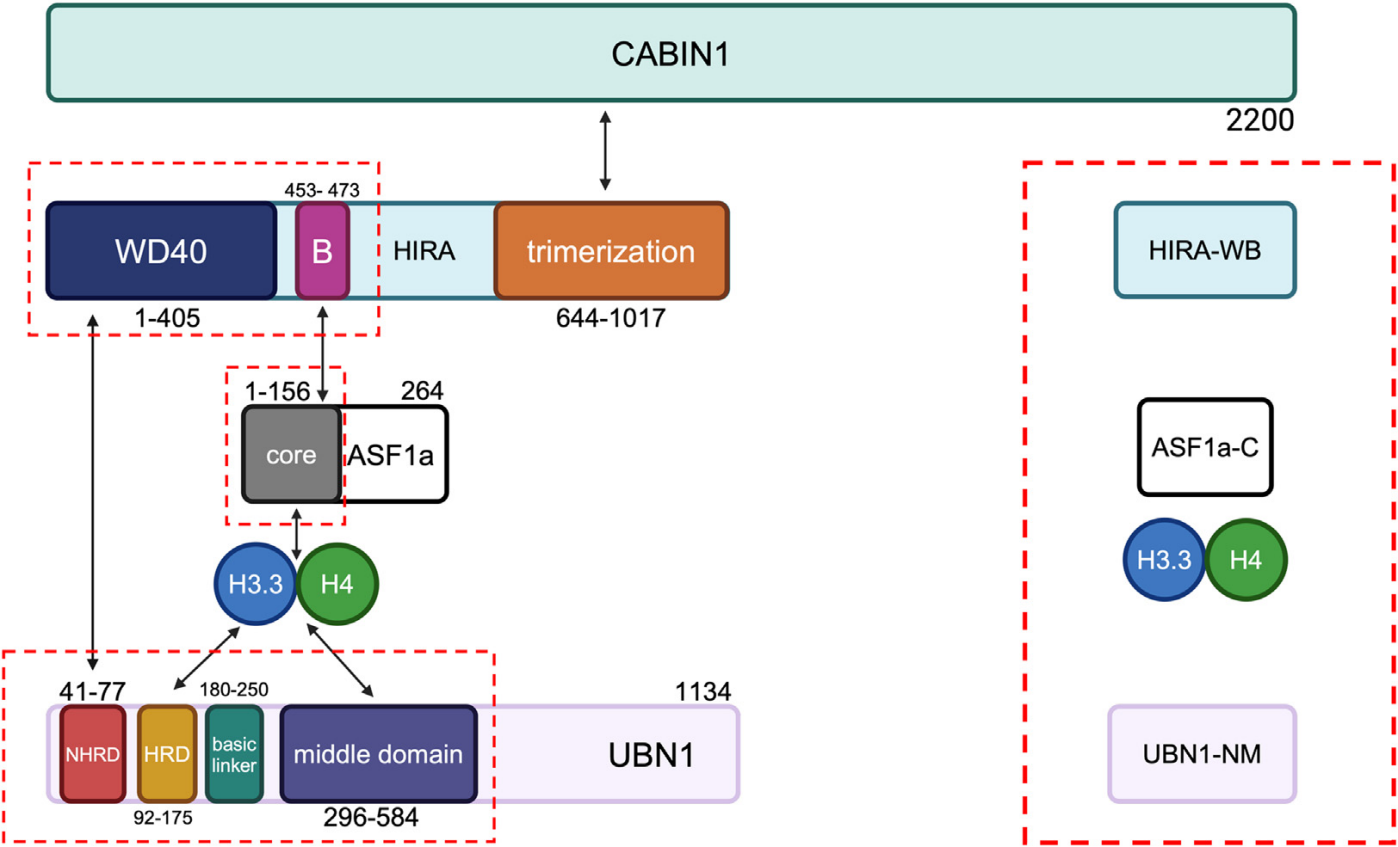
**Annotated domain map of all subunits of the human HIRA histone chaperone complex.** The specific constructs used throughout the study are highlighted by the *dotted red boxes* with their simplified notations indicated on the *right*.

The HIRA subunit has been shown to include three distinct domains - (1) a WD40 repeat domain which binds UBN1 with an apparent *K*_d_ in the nanomolar range (28), (2) a B-domain responsible for ASF1a binding (30), and (3) a C-terminal structured domain able to homotrimerize *in vitro* and in cells and binds with nanomolar affinity to two copies of the CABIN1 subunits *in vitro* (31) (Fig. 1). The functional role of the two CABIN1 subunits is largely unknown.

Here, we characterize ASF1a delivery of histones to the HIRA complex and the mechanism of ASF1a dissociation from HIRA by investigating several intermediate steps of the process. We show that a stable HIRA/UBN1 subcomplex can accommodate H3.3/H4 dimers or tetramers with equal affinity, it can bind ASF1a/H3.3/H4 in an energetically poised state, and that ASF1a dissociation from H3.3/H4 can be driven by competition with double-stranded DNA. Our observations support a model whereby H3.3/H4 tetramerization on DNA is the event that brings two HIRA/UBN1 assemblies together within the large, megadalton HIRA chaperone complex for histone deposition onto DNA.

## Results

### HIRA/UBN1 form a 1:1 subcomplex for ASF1a association

**Figure 2:**
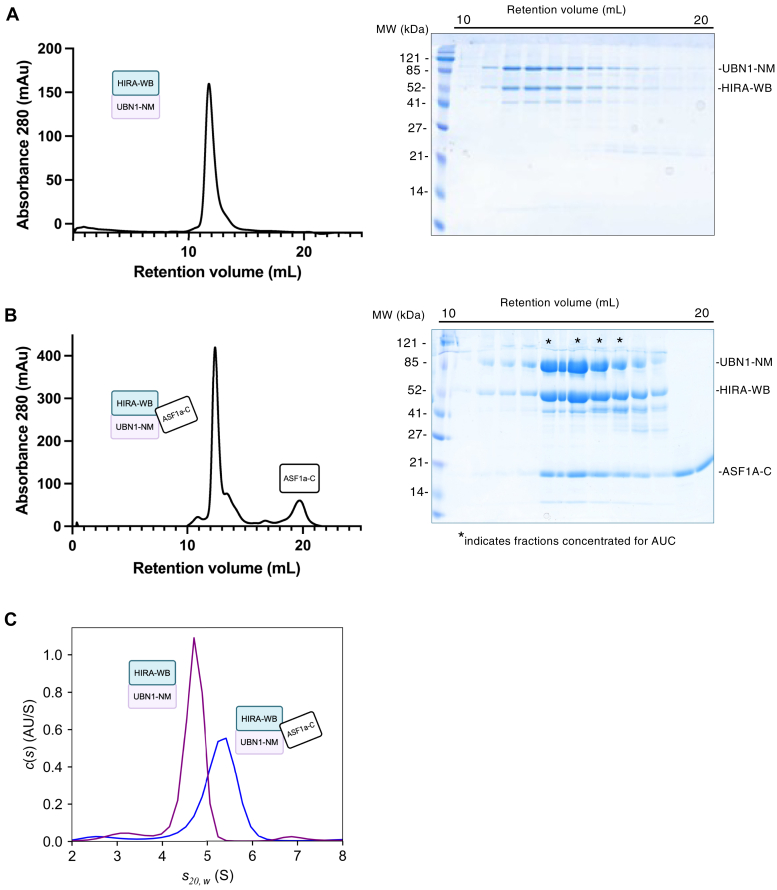
**Purification and characterization of monodisperse HIRA/UBN1/ASF1a complexes.***A*, SDS-PAGE analysis (*right*) of fractions from a representative purification of the HIRA/UBN1 complex by size exclusion chromatography (*left*) suggests a stoichiometric, monodisperse complex. *B*, SDS-PAGE analysis (*right*) of fractions from the final purification step of the HIRA-WB/UBN1-NM/ASF1a-C complex by size exclusion chromatography (*right*) suggests a stoichiometric, monodisperse complex. *C*, sedimentation velocity analytical ultracentrifugation (SV-AUC) of the respective peak fractions of the complexes confirm a single, monodisperse population of the HIRA-WB/UBN1-NM subcomplex (*blue*) and a single, monodisperse population of the HIRA-WB/UBN1-NM/ASF1a-C subcomplex (*purple*). Calculated molecular weights are consistent with 1:1 and 1:1:1 complexes, respectively.

The 3:2 stoichiometry seen in the HIRA C terminus:CABIN1 interaction (31) prompted us first to investigate the stoichiometry of the HIRA N terminus relative to its known interactors UBN1 and ASF1a. Specific constructs used throughout this study are shown in Figure 1. Co-expression of HIRA 1 to 473, containing the WD40 and B domains (herein referred to as HIRA-WB), and UBN1 1 to 584, containing the NHRD, HRD, basic linker and middle domain (herein referred to as UBN1-NM) in Sf9 cells yields a stable complex *in vitro* (Fig. 2*A*). To determine HIRA-WB:UBN1-NM stoichiometry, we ran sedimentation velocity analytical ultracentrifugation (SV-AUC) of peak fractions of the complex (Fig. 2*A*). SV-AUC demonstrates the formation of a heterodimer of HIRA-WB and UBN1-NM (Figs. 2*C*, S1, Table 1). The resolution of a single, monodisperse 129 kDa (mass integral range of 110–162 kDa) molecular weight assembly (theoretical molecular weight = 120 kDa) precludes the homodimerization of UBN1-NM through its middle domain that has been previously reported with protein recombinantly expressed in bacteria (26). This suggests that UBN1-NM’s association with HIRA-WB disfavors UBN1-NM dimerization.

**Table 1:** Summary of sedimentation velocity results

Protein	Loading concentrations (OD_280_)	Loading concentrations (μM)	Sedimentation coefficient (S20,W)	Frictional ratio (f/f0)	Experimentally measured mass (kDa)^a^	Theoretical mass (kDa)
HIRA-WB/UBN1-NM	0.27, 0.3, 0.64	2.01, 2.23, 4.77	4.7	1.92	129 (110–162)	120
HIRA-WB/UBN1-NM/ASF1a-C	0.397, 0.452	2.55, 2.90	5.2	1.65	120 (107–132)	139
HIRA-WB/UBN1-NM/H3.3/H4	0.2, 0.5, 0.7	2.06, 4.11, 6.16	4.7 and 8.05 Kd = 6 μM (68% CI 4–7 μM)	2.00	NA	147 and 294
HIRA-WB/UBN1-NM/ASF1a-C/H3.3/H4	0.4	2.41	6.1	1.59	162 (153–179)	166

a mass integral range is indicated in parenthesis.

To investigate the interaction of HIRA-WB/UBN1-NM with ASF1a, we prepared ASF1a residues 1 to 156, containing the core domain previously shown to be sufficient for binding histones and HIRA (27), herein referred to as ASF1a-C. We found that the 1:1 HIRA-WB:UBN1-NM stoichiometry is preserved upon the addition of ASF1a. SV-AUC experiments of peak fractions from the SEC containing HIRA-WB, UBN1-NM and ASF1a-C (Fig. 2*B*) also revealed a single, monodisperse assembly with a calculated molecular weight of 120 kDa (mass integral range of 107–132 kDa) (theoretical molecular weight = 139 kDa) and average sedimentation coefficient that was higher (5.2 *versus* 4.7) than the HIRA-WB/UBN1-NM assembly (Figs. 2*C*, S1, Table 1) which likely indicates formation of a stoichiometric heterotrimer with a distinct molecular shape from the HIRA-WB/UBN1-NM heterodimer. To further support a 1:1:1 HIRA-WB/UBN1-NM/ASF1-C complex we subjected both HIRA-WB/UBN1-NM and HIRA-WB/UBN1-NM/ASF1-C complexes to mass photometry, revealing molecular masses of 123 kDa and 134 kDa for both complexes, respectively (Fig. S2). Because the physiologically relevant interaction domains are preserved in these constructs, we conclude that the HIRA:UBN1:ASF1 stoichiometry within the HIRA complex is 1:1:1.

### HIRA/UBN1 multimerization is driven by H3.3/H4 tetramerization

**Figure 3:**
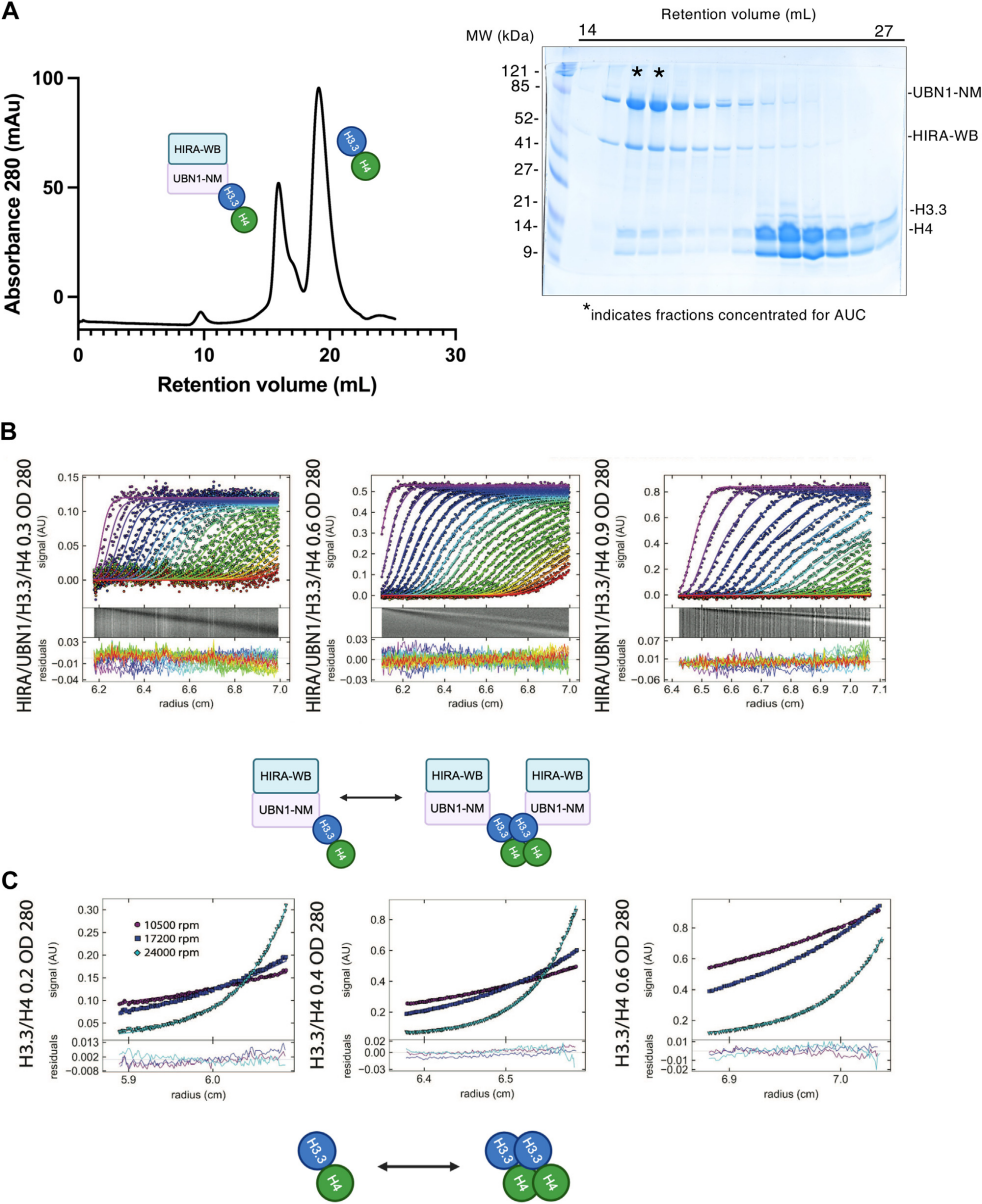
**The addition of H3.3/H4 to the HIRA/UBN1 subcomplex leads to the formation of two distinct species.***A*, SDS-PAGE analysis (*right*) of fractions from the size exclusion chromatography of HIRA-WB/UBN1-NM in the presence of excess H3.3/H4 (*left*) resolves into two peak fractions containing HIRA-WB/UBN1-NM/H3.3/H4 and H3.3/H4. *B*, global fit across SV-AUC analysis of the peak from A corresponding to the HIRA-WB/UBN1-NM/H3.3/H4 complex, run at three different loading concentrations suggests a monomer-dimer equilibrium in which a monomer is defined as a 1:1:1:1 assembly of HIRA-WB:UBN1-NM:H3.3:H4 with a dissociation constant of 6 μM. *C*, sedimentation equilibrium analytical ultracentrifugation (SE-AUC) of H3.3/H4 alone analyzing sedimentation of three different loading concentrations across three different speeds fits a monomer-dimer equilibrium model in which a monomer is defined as a H3.3/H4 heterodimer. The calculated dissociation constant is 6 μM.

The next question we sought to answer was the effect of histone binding on the HIRA-WB/UBN1-NM assembly. When we added excess H3.3/H4 to the HIRA-WB/UBN1-NM assembly, we observed two elution peaks corresponding to the HIRA-WB/UBN1-NM/H3.3/H4 and H3.3/H4 complexes (Fig. 3*A*). Fractions corresponding to the first peak were concentrated and analyzed by SV-AUC. Across multiple loading concentrations, we consistently observed two distinct species (Fig. 3*B*, Table 1) most likely indicative of a 1:1:1:1 HIRA-WB:UBN1-NM:H3.3:H4 complex and a higher order multimer of this complex. Global fit of the SV-AUC data fit best to a monomer-dimer equilibrium model (χ^2^ = 0.67) in which a monomer is defined as a 1:1:1:1 assembly of HIRA-WB:UBN1-NM:H3.3:H4 (theoretical molecular weight = 147 kDa) and a dimer is defined as a 2:2:2:2 assembly of HIRA-WB:UBN1-NM:H3.3:H4 (theoretical molecular weight = 294 kDa). The calculated *K*_d_ of the HIRA:UBN1:H3.3:H4 complex dimerization was determined to be 6 μM (68% CI 4-7 μM) (Fig. 3*B*, Table 1). Because the second species formation is only observed in the presence of histones and not in the presence of HIRA-WB/UBN1-NM alone, we hypothesized that histone tetramerization likely drives the dimerization of this assembly.

To further investigate this possibility, we ran sedimentation equilibrium analytical ultracentrifugation (SE-AUC) of H3.3/H4 alone and obtained data that fit a monomer-dimer equilibrium (χ^2^ = 0.35) (Fig. 3*C*, Table 2). In these data, each H3.3/H4 heterodimer is considered a monomer in the model. The calculated *K*_d_ = 6 μM (68% CI 2–14 μM) therefore describes H3.3/H4 tetramerization under these buffer conditions. Because this value matches the 6 μM *K*_d_ calculated when the histones were pre-bound to HIRA-WB/UBN1-NM, we conclude that histone tetramerization drives the association of two HIRA/UBN1 units in the context of the HIRA complex.

**Table 2:** Summary of sedimentation equilibrium results

Protein	Loading concentrations (OD_280_)	Loading concentrations (μM)	Rotor speeds (rpm)	Model fit	Theoretical mass (kDa)	Global fit X^2^
H3.3/H4	0.3, 0.6, 0.9	4 μM, 10 μM, 25 μM	15,000, 25,000, 30,000	Monomer-Dimer	27 (dimer) 54 (tetramer)	0.35

### ASF1a blocks HIRA/UBN1 subcomplex dimerization by blocking H3.3/H4 tetramerization

**Figure 4:**
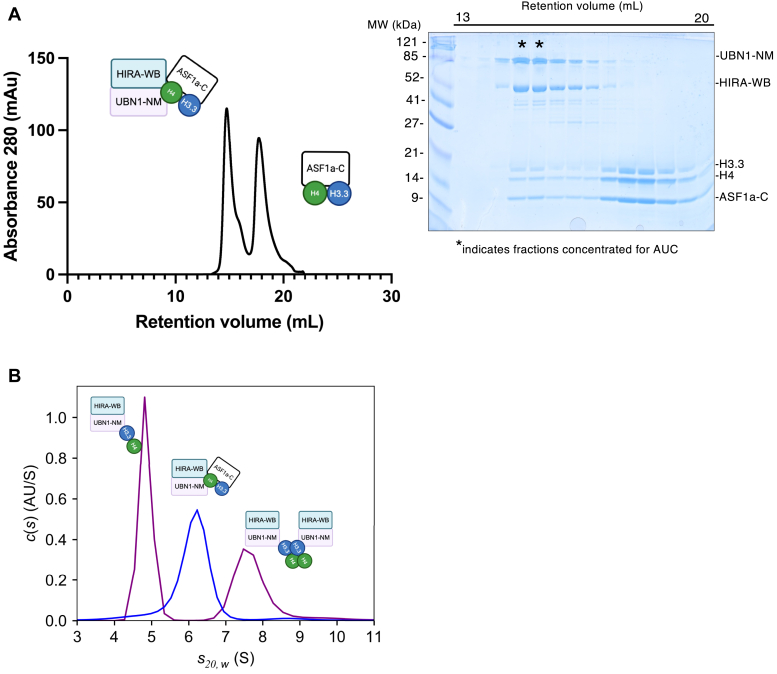
**The addition of ASF1a/H3.3/H4 to HIRA/UBN1 forms a pentameric complex.***A*, SDS-PAGE analysis (*right*) of size exclusion chromatography of the HIRA-WB/UBN1-NM in the presence of excess ASF1a-C/H3.3/H4 complex forms a monodisperse HIRA-WB/UBN1-NM/ASF1a-C/H3.3/H4 complex (*left*). *B*, SV-AUC of peak fractions containing the HIRA-WB/UBN1-NM/ASF1a-C/H3.3/H4 complex reveals a single population with a predicted molecular weight greater than that of the HIRA-WB/UBN1-NM/H3.3/H4 monomer but less than that of the HIRA-WB/UBN1-NM/H3.3/H4 dimer.

Because ASF1a is known to bind to the H3.3/H4 tetramerization interface, we hypothesized that the addition of ASF1a would disrupt the observed monomer-dimer equilibrium of the HIRA-WB/UBN1-NM/H3.3/H4 interaction. Indeed, upon the addition of excess ASF1a-C/H3.3/H4 to HIRA-WB/UBN1-NM and subsequent gel filtration chromatography, two peaks corresponding to HIRA-WB/UBN1-NM/ASF1a-C/H3.3/H4 and ASF1a-C/H3.3/H4 complexes were observed (Fig. 4*A*). SV-AUC of fractions from the first peak reveals a single species with a mass of 162 kDa (mass integral range of 153–179 kDa), with an intermediate sedimentation coefficient between 1:1:1 and 2:2:2 HIRA-WB/UBN1-NM/H3.3/H4 complexes (Fig. 4*B*), indicative of a heteropentamer of HIRA-WB/UBN1-NM/ASF1a/H3.3/H4 (theoretical molecular weight = 170 kDa). This data supports the conclusion that physiologically relevant tetramerization of two H3.3/H4 dimers drives the association of the two HIRA-WB/UBN1-NM complexes to which they are bound. This also confirms our structure-based inference that one H3.3/H4 dimer can be bound to both HIRA/UBN1 and ASF1a simultaneously in the context of a poised intermediate complex.

### The heteropentameric HIRA/UBN1/ASF1a/H3.3/H4 complex appears poised for histone deposition

**Figure 5:**
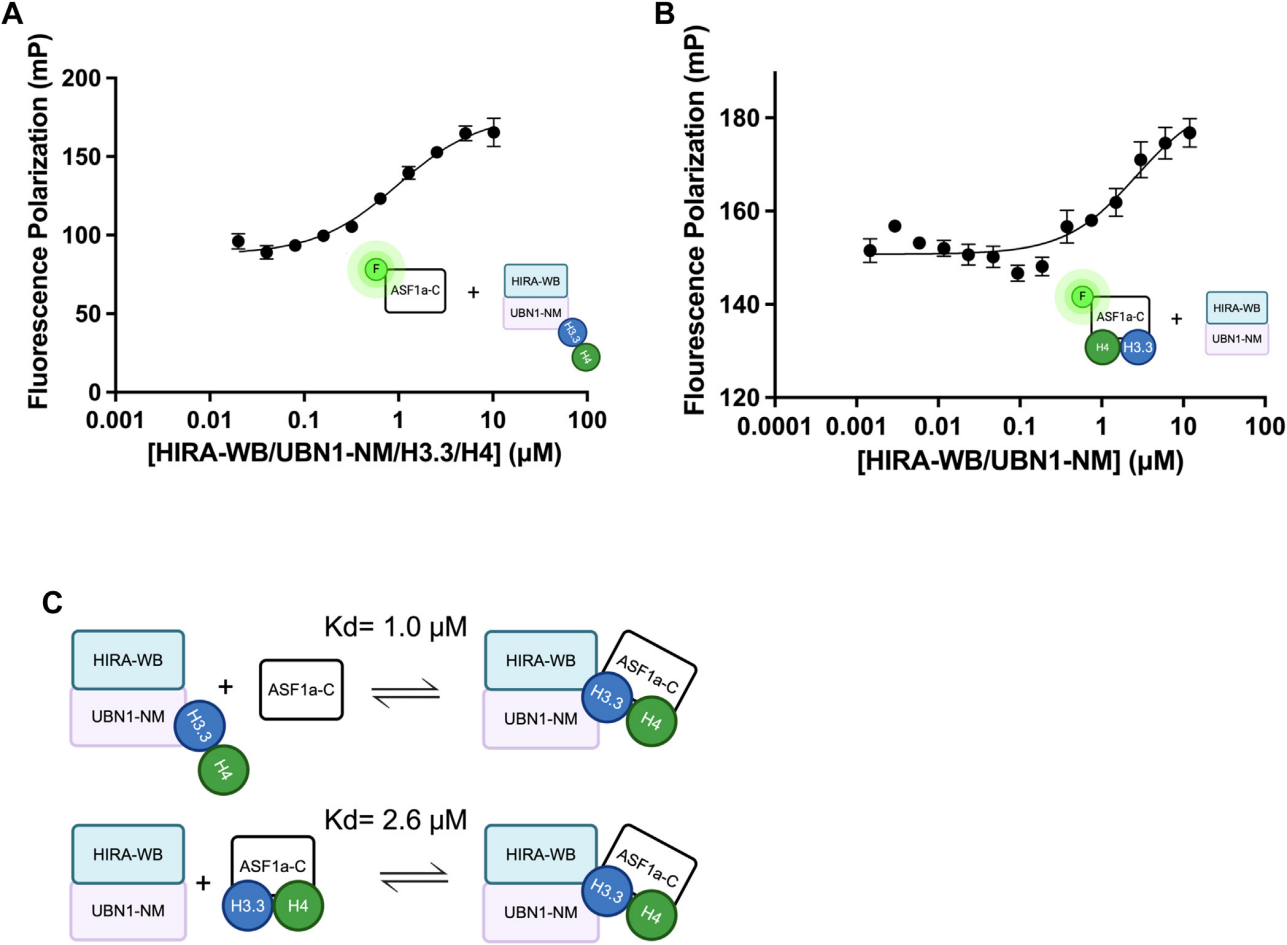
**Histones decrease the affinity of ASF1a for the HIRA/UBN1 subcomplex.***A* and *B*, ASF1a-C binds the HIRA-WB/UBN1-NM/H3.3/H4 complex with a *K*_d_ of 1.0 μM (*A*) that is weakened by about 2.5 fold to 2.6 μM (*B*) when histones are instead bound to ASF1a-C, suggesting that histones destabilize Asf1 binding to HIRA-WB/UBN1-NM. Error bars shown represent the SEM of five independent replicates. *C*, interactions between ASF1a and HIRA/UBN1 in the presence and absence of histones are summarized from most to least favorable.

Given that ASF1a-C/H3.3/H4 associates with HIRA-WB/UBN1-NM in a stable heteropentameric assembly, we then asked how ASF1a is ultimately released from this complex to allow histone tetramerization and deposition to proceed. To understand this process, we used fluorescence polarization binding assays to characterize the binding affinities of ASF1a-C (±H3.3/H4) for HIRA-WB/UBN1-NM. We hypothesized that the relative strengths of these binding affinities would help clarify the biophysical role of this intermediate assembly in the context of the histone deposition mechanism. We incubated ASF1a-C with an AlexaFluor-488 tetrafluorophenyl (TFP) ester to achieve a degree of labeling of 1.5 and then titrated in increasing concentrations of HIRA-WB/UBN1-NM/H3.3/H4 to generate a complete binding curve with a calculated a *K*_d_ of 1.0 ± 0.2 μM (Table 3) using a one site total binding model (Fig. 5*A*). Instead, when HIRA-WB/UBN1-NM/H3.3/H4 was titrated into fluorescently labeled ASF1a-C preassembled with H3.3/H4, we calculated a *K*_d_ of 2.6 ± 1.0 μM (n = 5) (Table 3) using a one site binding model (Fig. 5*B*). The larger dissociation constant of ASF1a-C for HIRA-WB/UBN1-NM in the presence of prebound histones indicates that pre-bound histones destabilize the interaction between ASF1a-C and HIRA-WB/UBN1-NM. This suggests that the HIRA/UBN1/ASF1a/H3.3/H4 heteropentameric complex may represent a poised state from which ASF1a is primed to ‘hand off’ histones to HIRA/UBN1, coupled with ASF1 eviction and histone tetramerization and deposition (Fig. 5*C*).

**Table 3:** Summary of fluorescence polarization results

Figure	Protein, protein complex, or DNA binder	Labeled probe	Kd or Ki value ± SD (μM)^a^	Number of independent Replicates^b^
5A	HIRA-WB/UBN1-NM/H3.3/H4	10 nM ASF1a-C	1.0 ± 0.2 (Kd value)	5
5B	HIRA-WB/UBN1-NM	10 nM ASF1a-C bound to unlabeled H3.3/H4	2.6 ± 1.0 (Kd value)	5
6A	H3.3/H4	dsDNA	0.24 ± 0.03 (Kd value)	5
6A	ASF1a-C/H3.3/H4	dsDNA	1.1 ± 0.14 (Kd value)	5
6B	H3.3/H4 bound to DNA	10 nM ASF1a-C	Not determined	5
6C	60 bp dsDNA	10 nM ASF1a-C bound to unlabeled H3.3/H4	0.28 ± 0.20 (Ki value)	3
6D	HIRA-WB/UBN1-NM/H3.3/H4	dsDNA	0.04 ± 0.01 (Kd value)	7
6D	HIRA-WB/UBN1-NM	dsDNA	0.37 ± 0.06 (Kd value)	7
6D	HIRA-WB/UBN1-NM/ASF1a-C	dsDNA	0.97 ± 0.13 (Kd value)	7
Supplemental 3	H3.3/H4	10 nM ASF1a-C	0.28 ± 0.05 (Kd value)	3

a ± values represent the standard deviation of the Kd or Ki values.

b Error bars for each fluorescence polarization curve represent the SEM for each set of independent replicates.

### DNA competes for H3.3/H4 away from ASF1a while the HIRA/UBN1 subcomplex appears to perform a scaffolding role

**Figure 6:**
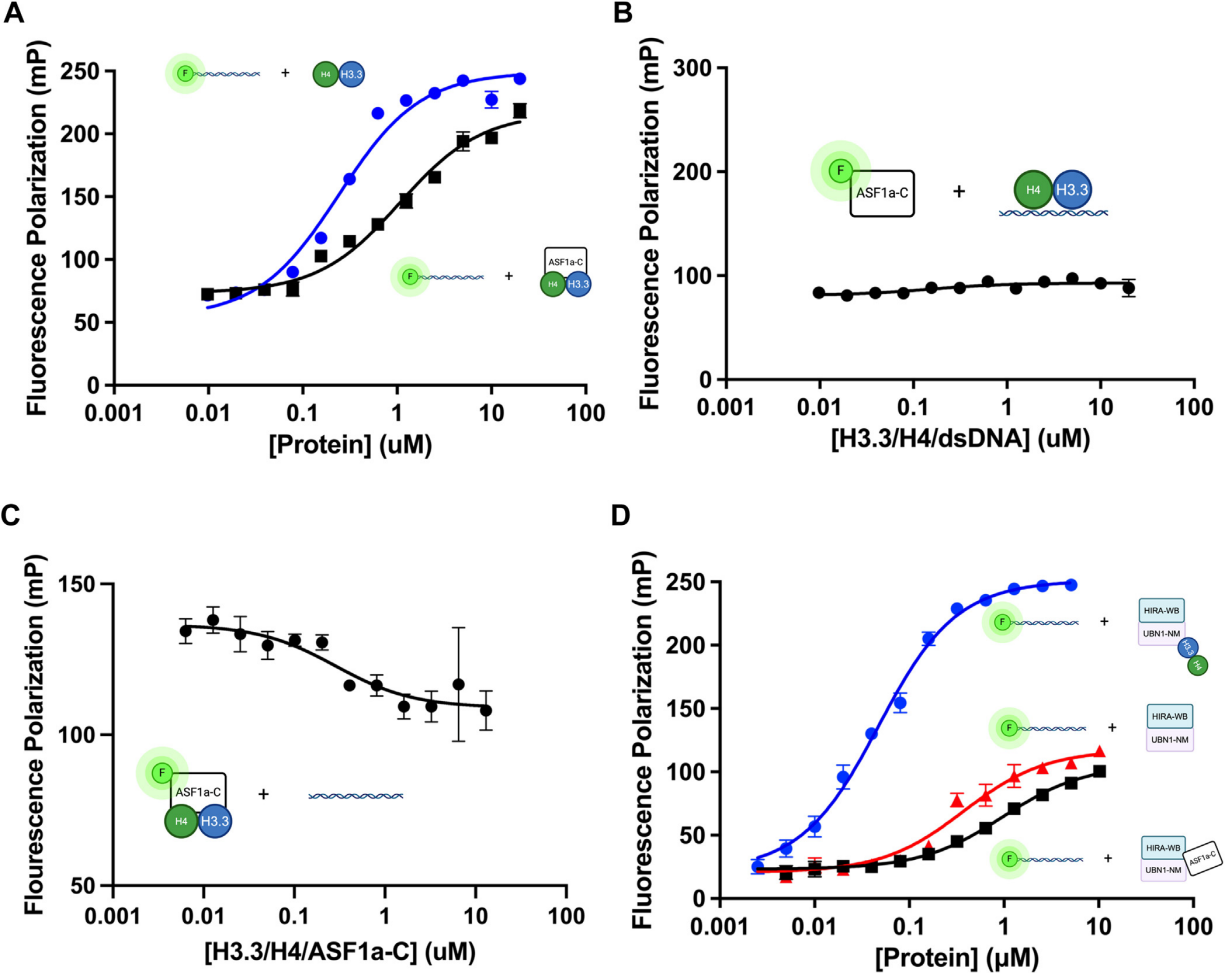
**DNA competes histones away from ASF1a.***A*, Addition of fluorescently labeled DNA to ASF1a-C/H3.3/H4 reveals a complete binding curve with a calculated *K*_d_ of 1.0 μM. Error bars shown represent SEM of five independent replicates. *B*, when fluorescently labeled ASF1a-C is added to DNA/H3.3/H4, no binding is observed. *C*, DNA competes H3.3/H4 away from ASF1a-C with an inhibition constant of 280 nM. Error bars shown represent the SEM of three independent replicates. *D*, DNA binds a HIRA-WB/UBN1-NM/H3.3/H4 complex with a Kd of 40 nM and in a manner that is highly dependent on the presence of H3.3/H4 within the complex. Error bars shown represent SEM of seven independent replicates.

The actual dissociation of ASF1a from the poised HIRA/UBN1/ASF1a/H3.3/H4 complex still must be facilitated by a factor with a greater affinity for histones than has been shown for any part of the HIRA complex. The most likely factor for this is double-stranded DNA which has been shown to bind to histones with higher affinity (*K*_d_ = 1.6 nM for 146 bp DNA) (29) than full-length ASF1a (*K*_d_ = 2.5 nM) (30) or UBN1 (*K*_d_ = 7 μM) (27). We first titrated H3.3/H4 into a 40-mer of fluorescently labeled double-stranded DNA and determined the *K*_d_ of the interaction to be 0.24 ± 0.03 μM (n = 5) in our system (Fig. 6*A*, Table 3). This is the strongest interaction we have characterized so far, and we sought to determine how the presence of ASF1a would affect it.

We next titrated ASF1a-C/H3.3/H4 into fluorescently labeled DNA and observed an increase in fluorescence polarization indicative of DNA binding, with a calculated *K*_d_ of 1.1 ± 0.1 μM (Fig. 6*A*, Table 3) but it was not immediately obvious whether DNA was binding to the ASF1a-C/H3.3/H4 complex or competing ASF1a away and binding histones alone. When we titrated DNA-bound histones into fluorescently labeled ASF1a-C, we saw no evidence of ASF1a-C binding (Fig. 6*C*), which suggests that ASF1a does not bind DNA-engaged histones even though the ASF1a and DNA binding interfaces are distinct. These observations were further supported by a competition assay in which labeled ASF1a-C was pre-bound to a concentration of H3.3/H4 where ∼ half of ASF1a-C is saturated with H3.3/H4 (the Kd of the interaction as determined in Fig. S3 and Table 3) and increasing concentrations of dsDNA were titrated into the solution. The signal decrease in fluorescence polarization indicates that dsDNA competes ASF1a off of H3.3/H4 with a Ki of 0.28 ± 0.2 μM (Fig. 6*D*, Table 3). Together, these data demonstrate that DNA competes histones off of the HIRA/UBN1/ASF1 complex.

To directly evaluate histone binding in the context of the HIRA-WB/UBN1-NM/H3.3/H4 complex, we titrated this complex, as well as control complexes lacking histones or ASF1a-C in place of histones. We observed significantly higher DNA-binding capacity of the histone-containing complex (Kd = 40 nM, Table 3), relative to the complexes not containing histones. The DNA likely binds more strongly to the HIRA-WB/UBN1-NM/H3.3/H4 complex over free histones, due to the additional DNA binding affinity of UBN1 as we previously demonstrated (29). Taken together, based on these data, we conclude that dsDNA plays a key role in ASF1a dissociation in the context of histone deposition by the HIRA complex.

## Discussion

**Figure 7:**
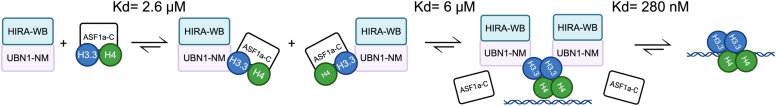
**Model for H3.3/H4 deposition by the HIRA complex.** We propose that the formation of two distinct HIRA/UBN1/ASF1a/H3.3/H4 heteropentamers within close proximity to each other and double-stranded DNA are poised for a concerted release of ASF1a driven by histone tetramerization and the high affinity of histones for DNA.

In this study we focused on the regions of the HIRA complex that most likely directly contribute to ASF1a-assisted deposition of the (H3.3/H4)_2_ tetramer on DNA. Biochemical and biophysical studies to probe protein stoichiometry and protein/DNA binding properties support a model in which ASF1a delivers histone H3.3/H4 dimers to a HIRA/UBN1 complex, H3.3/H4 tetramerization drives the association of two HIRA/UBN1 subunits, and histone binding to DNA drives release of ASF1a and subsequent histone deposition. A schematic model for this is illustrated in Figure 7.

The full-length HIRA subunit contains a C-terminal homotrimerization domain not included in this study which theoretically could facilitate the presentation of two copies of the ASF1a/H3.3/H4 for deposition of a (H3.3/H4)_2_ tetramer. Arguing against this possibility, however, is the observation that the orthologous HIRA complex from *Saccharomyces cerevisiae* (Hir complex) contains an analogous heterotrimer containing one copy of Hir1 and two copies of Hir2 (32) (Fig. S4). In this complex, only Hir1 contains a conserved B domain (32) so there is only one ASF1a-coordinating B-domain per Hir(1/2) trimer. This suggests that the C-terminal trimerization of the HIRA subunit is likely not required to coordinate the association of two copies of ASF1a/H3.3/H4 to facilitate histone tetramerization in the human system. Furthermore, in both the yeast and human systems, between the HIRA/Hir(1/2) N-terminal domain that binds UBN1 and ASF1a used in this study and the C-terminal trimerization domain is a several hundred residue region that contains a predicted disordered region (Fig. S4) and is therefore unlikely to play a role in positioning the N termini in a mechanistically relevant way. An attractive hypothesis that is supported by a recently reported cryo-EM structure of the orthologous Hir complex from *S. cerevisiae* is that a dimer of Hir/Hi2 trimers mediates histone deposition on DNA (33).

The structural similarity between the C-terminal trimerization domain of HIRA and the yeast replisome component Ctf-4 has recently been highlighted (34) and suggests the role of this domain could be more architectural than functional in the context of the intact HIRA complex. Cryo-EM structures have shown that the C-terminal trimerization domain of Ctf-4 coordinates binding to two copies of CMG helicase and one Pol α-primase at the replication fork. The conserved domain in HIRA, therefore, may play a scaffolding role in coordinating interactions with nuclear complexes such as RNA polymerase (35) during transcription, RPA (36) during DNA repair, or the SRCAP complex (37) for histone H2A.Z deposition at poised genes in mouse embryonic stem cells. This hypothesis is also supported by the recently reported cryo-EM *S. cerevisiae* Hir structure (33).

The data presented here suggest that the HIRA complex is unique among H3/H4 chaperones in that upon receipt of H3.3/H4 dimers from ASF1a it requires neither chaperone-chaperone nor chaperone-DNA interactions to facilitate H3.3/H4 tetramerization and deposition. The crystal structure of ASF1a/H3.3/H4 bound to the HRD of UBN1 indicated that histones could be accommodated by both chaperone subunits simultaneously (27), but here we show evidence that confirms this association in the context of a larger portion of the HIRA complex. We show that a 124 kDa HIRA/UBN1 subcomplex can accommodate not only ASF1a-chaperoned H3.3/H4 dimers but also (H3.3/H4)_2_ tetramers.

Our data also challenges previously hypothesized functions of the UBN1 subunit as we conclude that UBN1 homodimerization through its middle domain does not occur in the context of the HIRA/UBN1 subcomplex and therefore does not play a direct role in histone tetramerization. Instead, we propose that histone tetramerization and DNA engagement drive the association of two HIRA/UBN1/histone subcomplexes and ASF1a eviction, respectively, during tetrasome formation (Fig. 7). Structures of the HIRA complex in intermediate states interacting with ASF1a, histones, and DNA will be required to refine further mechanistic details underlying histone deposition by the HIRA complex.

## Experimental procedures

### Generation of expression plasmids

DNA constructs encoding HIRA 1 to 473 and UBN1 1 to 584 were PCR amplified from existing cDNA templates (28) and ligated into a pFastBac Dual vector (Invitrogen) with an N terminal His tag on HIRA. The GST-ASF1a/H3.3/H4 expression plasmid used was a previously described modification (27) of the GST-Asf1/H3.1/H4 polycistronic *E. coli* co-expression construct that was used for crystallization and structure determination (26). DNA constructs encoding H3.3 and H4 were PCR amplified from human and *X. laevis* cDNA, respectively, and ligated into a custom-engineered pET-Duet *E. coli* expression vector (Novagen) with an N-terminal His tag cleavable by TEV protease.

### Protein expression and purification

Sf9 cells were infected with high-titer baculovirus to co-express His-HIRA 1 to 473 and UBN1 1 to 584. Infected cells were suspended in 20 mM HEPES, pH 6.95, 500 mM NaCl, 5 mM BME, 10 mM imidazole, 0.1 mg/ml PMSF, EDTA-free complete protease inhibitor, lysozyme, and DNase and lysed by sonication. Lysate was clarified by centrifugation and the supernatant was subjected to nickel affinity chromatography to isolate the His-tagged HIRA-WB/UBN1-NM complex. Proteins were eluted with 300 mM imidazole and then subjected to heparin affinity chromatography on a 1 ml HiTrap Heparin HP affinity column. Fractions containing the HIRA-WB/UBN1-NM complex were pooled and concentrated alone or with ASF1a and/or H3.3./H4 in 50 kDa MWCO spin concentrators. Concentrated protein complexes were subjected to size exclusion chromatography on either a Superdex 200 or Superdex six increase column equilibrated with a buffer containing 20 mM HEPES, pH 6.95, 500 mM NaCl, and 0.5 mM TCEP.

The GST-ASF1a/H3.3/H4 complex was expressed in *E. coli* and purified as previously described (27)- briefly, the complex was expressed in BL21-Gold(DE3) cells (Agilent) induced with 0.8 mM IPTG, and protein was expressed for 8 h at 28 °C. Cell pellets were resuspended in lysis buffer containing 20 mM Tris pH 8.0, 500 mM NaCl, 1 mM PMSF, and 5 mM BME and lysed by sonication. Lysate was clarified by centrifugation and the supernatant was incubated with glutathione agarose resin (Gold Biotechnology) for 1 h, washed with buffer containing 20 mM Tris pH 8.0, 500 mM NaCl and 5 mM BME, and the Asf1/H3/H4 complex was then released from the resin with on-column cleavage using precision protease. The cleaved Asf1/H3/H4 complex was eluted in buffer containing 20 mM Tris pH 8.0, 500 mM NaCl, and 1 mM TCEP. The free Asf1/H3/H4 complex was then concentrated using a 3 K cutoff centrifugal filter device (Millipore) before a final gel filtration purification step with a HiLoad 16/600 Superdex S75 pg column (GE Healthcare). The ASF1a/H3.3/H4 complex and the excess ASF1a were pooled and concentrated, respectively. Histone proteins H3.3 and H4 were expressed in inclusion bodies in BL21-Gold(DE3) cells (Agilent). The histone proteins were purified separately and were refolded together as described previously (38).

Additional ASF1a/H3.3/H4 complex was assembled by combining twofold molar excess ASF1a with H3.3/H4 in 20 mM HEPES, pH 6.95, and 2M NaCl dialyzed overnight at 4 °C into 20 mM HEPES, pH 6.95, and 1M NaCl. The soluble complex was then subjected to size exclusion chromatography on a Superdex 75 increase column equilibrated with 20 mM HEPES, pH 6.95, 500 mM NaCl, and 0.5 mM TCEP.

### Analytical ultracentrifugation

Analytical ultracentrifugation studies were performed with an XL-I analytical ultracentrifuge (Beckman-Coulter) and a TiAn60 rotor with six channels for sedimentation equilibrium (SE) or two channels for sedimentation velocity (SV) Epon charcoal-filled centerpieces and quartz windows.

SV data were collected as absorbance profiles at 280 nm every 5 min for ∼9 h at 42,000 rpm at 4 °C (see Table 1) with absorbance step sizes of 0.001 cm. All protein complexes were prepared in a buffer containing 20 mM HEPES pH 6.95, 500 mM NaCl, and 0.5 mM TCEP. For all analyses, partial specific volume, solvent density, and viscosity were determined by chemical composition using SEDNTERP (39). Sedimentation velocity data for the HIRA-WB/UBN1-NM, HIRA-WB/UBN1-NM/ASCF1a-C, and HIRA-WB/UBN1-NM/ASCF1a-C/H3.3/H4 complexes were analyzed in Sedfit version 16.1c using a continuous c(s) distribution model; a confidence level of 0.68; and fitting for the baseline, RI noise, TI noise, and the meniscus (40).

For the HUHH (HIRA-WB/UBN1-NM/H3.3/H4) complex, where multiple peaks were observed, data was initially processed in Sedfit as described for the other complexes before being exported for global data analysis in Sedphat version 15.2 b (41). In Sedphat, the HUHH complex sedimentation velocity data was fit to a monomer-dimer self-association equilibrium model (42).

SE data were collected for the histone complex at 4 °C with detection at 247 nm for three loading concentrations (OD_247_ = 0.3, 0.6, 0.9.) Histones were prepared in a buffer containing 20 mM HEPES pH 7.5, 100 mM NaCl, 100 mM KCl, 2 mM MgCl_2,_ 1 mM EDTA, and 0.05% Brij-35. Experiments were run at 15,000 rpm, 25,000 rpm, and 30,000 rpm. Global analysis was performed on all sedimentation equilibrium and velocity data in Sedphat version 15.2 b using the monomer-dimer equilibrium model (Table 2) (41).

### Fluorescent probes

The FAM-labeled dsDNA used for the fluorescence polarization DNA-binding assays was assembled from synthesized DNA fragments (IDT). The sense sequence for the 10x ATGC probe is 5-FAM-ATGCATGCATGCATGCATGCATGCATGCATGCATGCATGC. The FAM-labeled sense fragment and unlabeled antisense fragment were dissolved in 10 mM Tris, pH 7.5, 10 mM NaCl, and 1 mM EDTA and were mixed at a 1:1 sense: antisense ratio, incubated at 95 °C for 5 min, then were slowly cooled to room temperature to allow for annealing.

To generate labeled ASF1a for HIRA-WB/UBN1-NM binding assays, recombinantly produced ASF1a was dialyzed overnight at 4C into 1X PBS and was labeled using the Alexa Fluor 488 Microscale Protein Labeling Kit (Invitrogen). Briefly, 100 ug ASF1a was incubated with 100 mM sodium bicarbonate and a 19-fold molar excess of Alexa Fluor 488 tetrafluorophenyl (TFP) ester at room temperature for 15 min. Excess dye was removed using Zeba dye and biotin removal spin columns (Thermo Scientific) and the degree of labeling was determined using a Nanodrop 2000 Sectrophotometer (Thermo Scientific).

To generate labeled ASF1a for DNA binding competition assays, recombinantly produced ASF1a was incubated in 1X PBS with a ten-fold molar excess of fluorescein-5-maleimide (Thermo Scientific) overnight in the dark at 4C. The labeling reaction was then run through multiple iterations of a 5 ml HiTrap desalting column equilibrated with 20 mM HEPES, pH 7.5, 150 mM NaCl, and 0.5 mM TCEP to remove excess dye.

### Fluorescence polarization binding assays

Protein and DNA used for these assays were prepared in 20 mM HEPES, pH 7.5, 150 mM NaCl, 0.5 mM TCEP, 0.001% Tween-20, and 1 mg/ml BSA. Direct binding assays were conducted by titrating increasing concentrations of respective proteins into a buffer containing 10 nM fluorescent probe and monitoring the increase in fluorescence polarization to determine the *K*_d_ of the protein/probe interaction. A competition binding assay was also performed by titrating increasing concentrations of unlabeled DNA binder into fluorescently labeled protein complex (10 nM labeled ASF1a complexed with 300 nM unlabeled H3.3/H4) and monitoring the decrease in fluorescence polarization to determine the Ki of the probe/binder interaction. All FP data were collected in minimum triplicate with an excitation wavelength of 485 nm and an emission wavelength of 535 nm using a TECAN Spark plate reader and binding curves were fit using a one-site total binding model with GraphPad Prism version 9.4.1 for macOS. Error bars represent SEM (Table 3).

### Mass photometry

All data were collected using a TwoMP mass photometer (Refeyn) calibrated with bovine serum albumin (66 kDa), beta-amylase (224 kDa), and thyroglobulin (670 kDa). Movies were acquired for 3000 frames (60 s) using AcquireMP software and default settings. Final protein concentrations were empirically determined to achieve ∼50 binding events per second by diluting with 20 mM HEPES, pH 7.5, 150 mM NaCl, and 0.5 mM TCEP. Raw data were analyzed through DiscoverMP software (2022 R1) with histogram mode and mass plot. Automated Gaussian peak detection was applied to estimate the masses, molecule counts, and sigma.

## Data availability

All data is contained in the manuscript or available upon request to the corresponding author.

## Supporting information

This article contains supporting information.

## Conflict of interest

The authors declare that they have no conflicts of interest with the contents of this paper.
