# Supplementary material for: HIRA complex deposition of histone H3.3 is driven by histone tetramerization and histone-DNA binding
Source: J Biol Chem. 2024 Jul 24;300(9):107604. doi: 10.1016/j.jbc.2024.107604 (PMC11388340; doi:10.1016/j.jbc.2024.107604)
Supplement: Supporting Information [file mmc1.pdf]

Supporting Information

HIRA complex deposition of histone H3.3 is driven by histone tetramerization and histone-DNA binding

Austin Vogt[1,2,*], Mary Szurgot[1,2,3,*], Lauren Gardner[1,2], David C. Schultz[1], and Ronen Marmorstein[1,2,#]

1 Department of Biochemistry and Biophysics, Perelman School of Medicine at the University of Pennsylvania, Philadelphia, PA 19104, USA

2 Abramson Family Cancer Research Center, Perelman School of Medicine at the University of Pennsylvania, PA, 19104 USA

3 Graduate Group in Biochemistry and Molecular Biophysics, Perelman School of Medicine at the University of Pennsylvania, PA, 19104 USA

Contents **Page**

Contents	Page
Figure S1	3
Figure S2	4
Figure S3	5
Figure S4	6

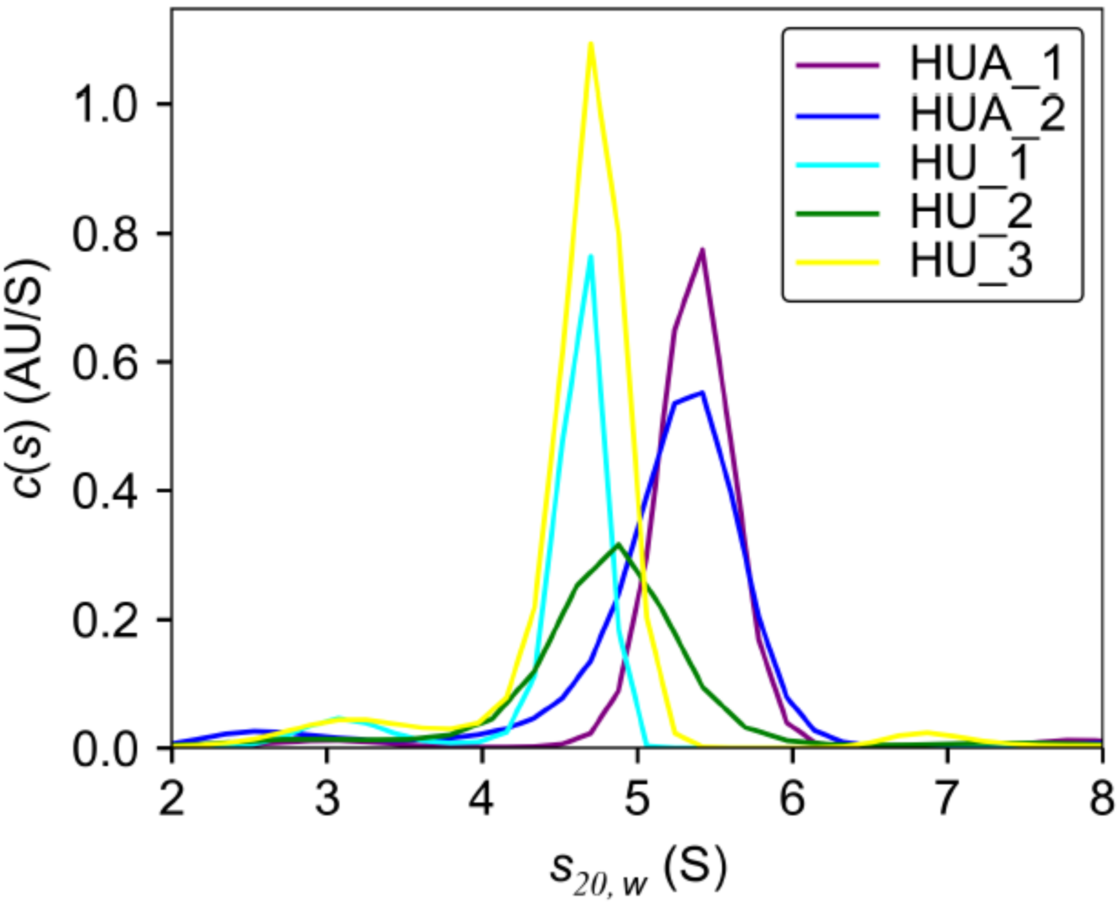

Supplemental Figure1: Sedimentation velocity analytical ultracentrifugation (SV-AUC) of individual runs of HIRA-WB/UBN1-NM and HIRA-WB/UBN1-NM/ASF1a-C subcomplexes. Individual runs are color-coded with HU representing HIRA-WB/UBN1-NM, and HUA representing HIRA-WB/UBN1-NM/ASF1a-C. The data shows that the HUA consistently has a higher sedimentation coefficient than HU.

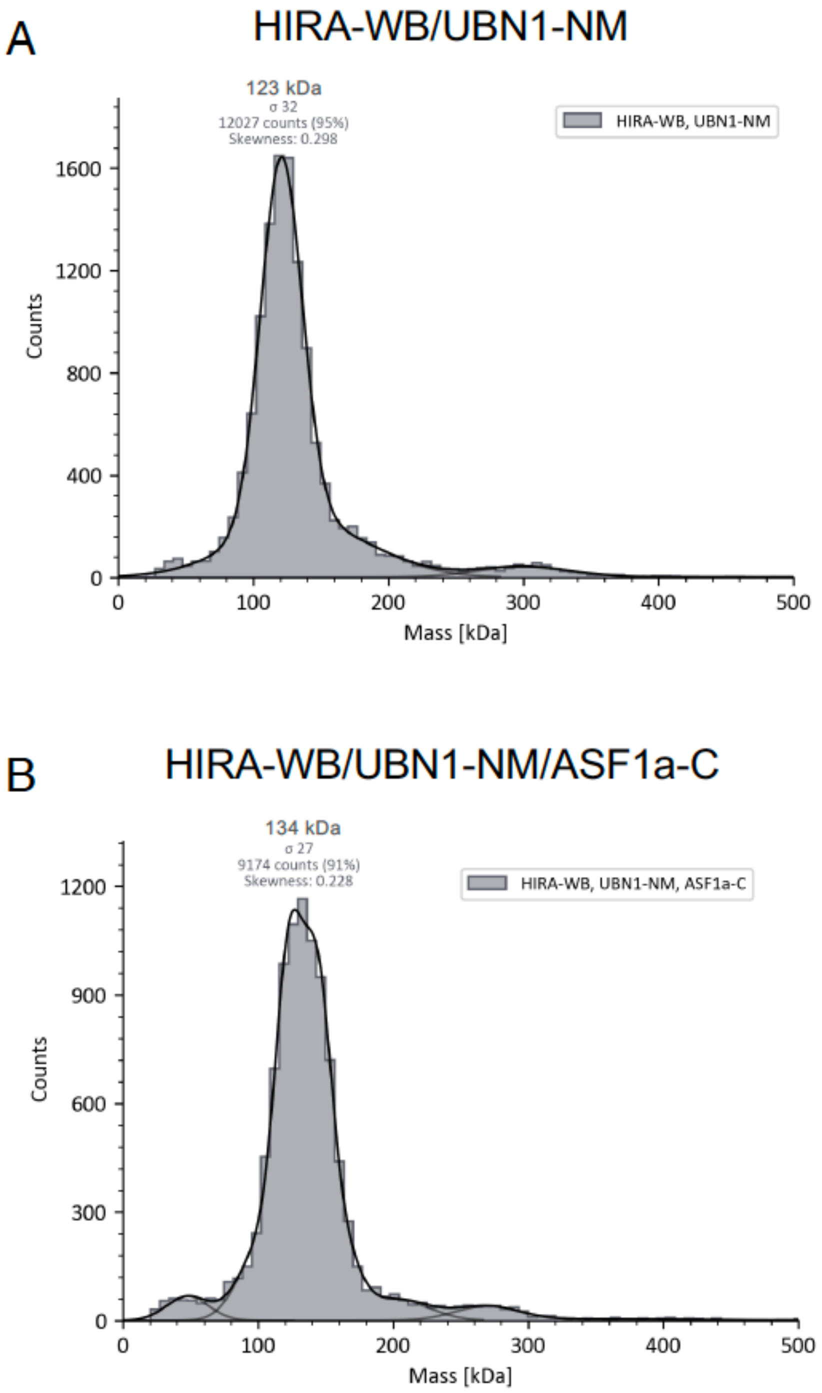

Supplemental Figure 2: Mass photometry of HIRA-WB/UBN1-NM and HIRA-WB/UBN1-NM/ASF1a-C subcomplexes. The molecular masses of complexes along with the counts and skewness values are indicated.

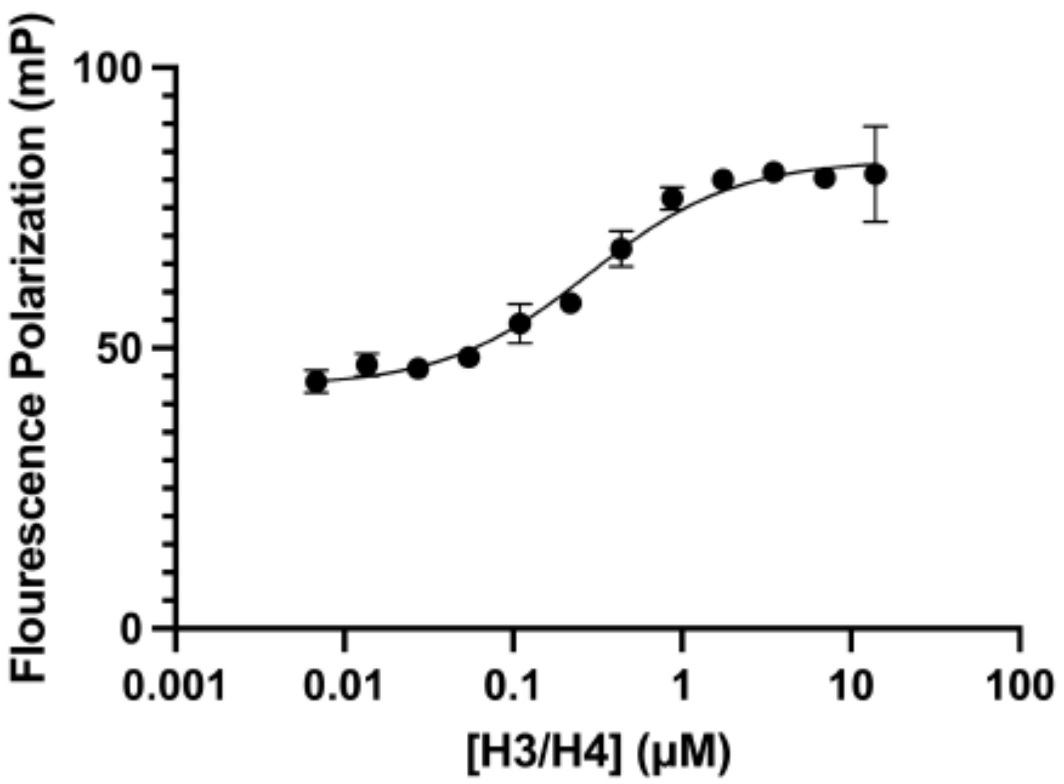

Supplemental Figure 3: Fluorescence polarization of H3.3/H4 titrated into labeled ASF1a-C. Experiments were carried out as described in figure 5 and yields a Kd value of 280 nM.

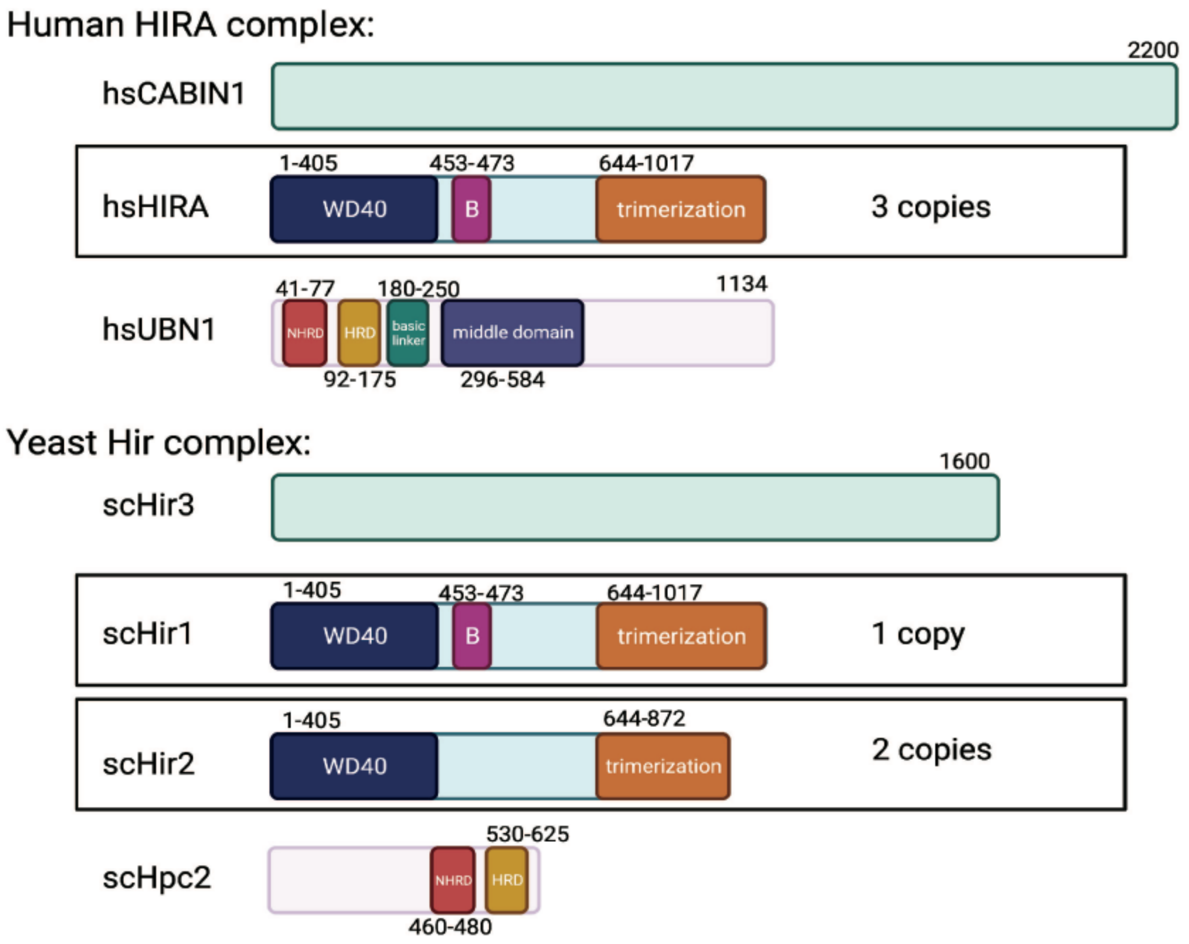

Supplemental Figure 4: The yeast Hir complex contains two distinct HIRA orthologs. The evolutionary conservation of only one B domain per Hir trimer in yeast suggests that only one ASF1a/H3.3/H4 is coordinated at a time by the Hir/HIRA complex.
